# Alkaline phosphatase is a predictor of Bone Mineral Density in postmenopausal females

**DOI:** 10.12669/pjms.35.3.188

**Published:** 2019

**Authors:** Sundus Tariq, Saba Tariq, Khalid Parvez Lone, Saba Khaliq

**Affiliations:** 1 *Dr. Sundus Tariq, MBBS, M.Phil. Associate Professor, Department of Physiology, University Medical & Dental College, Faisalabad- 38000, Pakistan., University of Health Sciences, Lahore, Pakistan*; 2 *Dr. Saba Tariq, MBBS, M. Phil. Associate Professor, Department of Pharmacology, University Medical & Dental College, Faisalabad- 38000, Pakistan*; 3 *Prof. Dr. Khalid Parvez Lone, M.Sc., M.I. Biol, Ph.D., FZSP. Ex-Professor and Head of Department Physiology/ Metabolic Disorders Government College University, Lahore, Pakistan*; 4 *Dr. Saba Khaliq, M.Sc. (GCU), Ph.D. (Pb), Post-doc. (Germany). Associate Professor, Department of Physiology, University of Health Sciences, Lahore, Pakistan*

**Keywords:** ALP, BMD, Postmenopausal females

## Abstract

**Objectives:**

The study was planned to determine whether serum calcium, phosphate and alkaline phosphatase (ALP) are predictors of bone mineral density (BMD) in postmenopausal non-osteoporotic, osteopenic, and osteoporotic females.

**Methods:**

In this cross sectional study, conducted at Shaikh Zayed Hospital, Lahore in the year 2014-2015, postmenopausal females between 50-70 years of age were taken and divided into three groups non-osteoporotic (n=52), osteopenic (n=69) and osteoporotic (n=47). Serum ALP, phosphate and calcium were used in a stepwise multiple regression analysis to predict T-score in these groups.

**Results:**

In normal postmenopausal females, the prediction model was statistically significant, F(2, 41) = 6.041, p < 0.05 and showed a T-score variance of 22%. T-score was primarily predicted by higher levels of phosphate and calcium. In postmenopausal osteopenic females, T-score was only predicted by lower levels of ALP. The model was statistically significant, F(1, 59) = 4.995, p < 0.05, and accounted for approximately 7% of the variance of T-score. In postmenopausal osteoporotic females, the prediction model contained no predictors.

**Conclusion:**

Our study suggested that calcium and phosphate are the strongest predictors of T-score in postmenopausal normal females, while in postmenopausal osteopenic females ALP was the strongest predictor of T-score. Elevated serum ALP levels may help in determining loss of BMD in postmenopausal females.

## INTRODUCTION

Menopause is an important risk factor that leads to bone loss in elderly women. During the whole life of women there is 50% loss of the trabecular and 30% of the cortical bone.^1^ Bone density and bone quality both are combined to form bone strength. This bone strength is difficult to measure in the hospital setting. However if there is no fracture, bone mineral density (BMD) is an alternate method to measure seventy percent of bone strength and is one of the important approaches to diagnose osteoporosis.^2^

Osteoporosis affects a huge number of people worldwide, and WHO consider this as one of the ten most serious global diseases. Osteoporosis is a chronic disorder and there are multiple factors that lead to osteoporosis. The basic defect is skeleton remodeling and decrease in bone mineral density that predispose person especially postmenopausal females to fragility fractures.^3^ Osteoporosis can lead to the development of hip fracture. Hip fracture is included in serious fractures and results in severe morbidity.^4^ The health complications of hip fractures are severe. There is loss of function, which is usually progressive in nature, and the patient is unable to move without support. These health consequences have led to mortality in up to 24% of women.^5^

A study in Pakistan identified the risk factors in postmenopausal women who had osteoporotic hip fractures and they identified that advancing age, early menopause, low BMI, longer duration of menopause, smoking, poor socioeconomic conditions, illiteracy, multiparity, lack of calcium supplements, injudicious use of steroids are the important risk factors.^6^

Alkaline phosphatase (ALP), a homodimeric protein with phosphorylating properties exist in many isozyme forms, the most common of them being tissue non-specific ALP. The two isoforms of tissue non-specific ALP, liver-specific ALP and bone-specific ALP (BALP) exists in almost equal proportion in serum.^7^ Physiologically, BALP adheres to osteoblastic cell membrane with only small amount released in serum. Its concentration in serum rises only in cases of increased remodeling of bone.^8^ The tissue mineralization stimulating effect of BALP is achieved mainly through inactivation of pyrophosphate and osteopontin, which are themselves mineralization inhibitors.^9^

Calcium and phosphate are important components of bony inorganic matrix and major factors in maintenance of bone health. Serum total ALP, osteocalcin, urinary calcium and hydroxyproline are strong predictors of bone loss.^10^ The deficiency of phosphate can also lead to bone pathology and clinical illness.^11^

The study was planned to determine and compare serum calcium, phosphate and alkaline phosphatase in three postmenopausal groups and to see whether these three parameters are predictors of BMD in postmenopausal non-osteoporotic, osteopenic, and osteoporotic females.

## METHODS

In this cross-sectional study, 168 postmenopausal females were selected and divided into three groups. Group A composed of 52 postmenopausal non-osteoporotic females (T-score ≥ -1.0). Age matched 69 postmenopausal osteopenic females were taken in group B (T-score -1.0 to -3.2). Group C was composed of 47 postmenopausal osteoporotic females (T score ≤ -3.2). The study population was selected after assessing 2500 females visiting orthopedics department of Shaikh Zayed Hospital, Lahore in the year 2014-2015. Required sample size was taken keeping in mind the inclusion and exclusion criteria. BMD and serum parameters including alkaline phosphatase, calcium and phosphate levels were carried out at postgraduate Physiology laboratory of University of Health Sciences, Lahore. Females between 50 to 70 years of age, having menopause for more than one year and required T-scores were included. Females with premature menopause, renal disease, liver disease, parathyroid problems and on medications like steroids, cyclosporine, oral contraceptives/ hormone replacement therapy and bisphosphonate therapy or any other medication affecting bone mineralization were excluded.^12^,^13^ Convenient sampling was used to select study population. Ethical approval to conduct the study was taken from University of Health Sciences, Pakistan’s ethical review committee.

Information from all the participants was obtained after taking informed consent. Anthropometric measures including waist circumference, hip circumference, height and weight were measured using standardized equipment. Body mass index (BMI) and waist to hip ratio (WHR) were calculated by using standard formulas. Bone mineral density (BMD) of postmenopausal females was measured using DBM Sonic Bone profiler manufactured by IGEA, Capri, Italy, Model: BP01.^14^ Three ml fasting blood was obtained in serum vacutainers. The sample was centrifuged for 10 minutes at 3000 revolutions per minute and serum was extracted and stored in aliquots at -40°C until used. Serum alkaline phosphatase, calcium and phosphate levels were determined by colorimetric method by using kits manufactured by Analyticon Biotechnologies AG. Germany, using a spectrophotometer with an inter assay coefficient of 1.5% and 1.3% respectively. Serum phosphate levels were determined by colorimetric method by using kit manufactured by Randox laboratories limited, UK. Using a spectrophotometer, with an interassay coefficient of 4.3%.

### Statistical Analysis

IBM-SPSS version 20 (Statistical Package for Social Sciences) was used for data analysis. Shapiro-Wilk’s statistics was used to check normality of data. Mean ± SD and median with IQR was given for normally and non-normally distributed quantitative variables respectively. Kruskal Wallis test was applied to compare quantitative variables between the three groups. Stepwise multiple regression analysis was performed to predict bone mineral density (T score). P-value ≤ 0.05 was taken as statistically significant.

## RESULTS

General characteristics of the study population are given in Table-I.

**Table-I:** General characteristics of study groups.

Parameters	Group A*(n=52)	Group B**(n=69)	Group C***(n=47)
Age (Years)^#^	52.50 (50 – 57)	58.00 (54 – 63)	60.00 (56 – 64)
Menopausal age (years)	49.59 (3.36)	49.42 (3.94)	48.36 (4.46)
Height (cm)	154.04 (5.63)	153.35 (4.59)	153.51 (5.69)
Weight (kg)	73.10 (13.25)	72.32 (13.69)	66.96 (12.32)
BMI (kg/m^2^)	30.76 (5.05)	30.87 (5.70)	28.37 (4.73)
Waist circumference (cm)	98.46 (9.85)	100.46 (9.94)	98.43 (9.98)
Hip circumference (cm)	108.71 (10.33)	109.70 (12.05)	103.68 (10.34)
Waist to hip ratio	0.90 (0.06)	0.91 (0.05)	0.94 (0.07)

* Group A = Postmenopausal non-osteoporotic females

** Group B = Postmenopausal osteopenic females

*** Group C = Postmenopausal osteoporotic female Values are given as Mean (SD).

# Value is given as Median (IQR).

On comparison of three groups, serum calcium levels were found to be significantly different (*p* < 0.001), while no significant difference was seen in serum phosphate and ALP between the three groups (Table-II).

**Table-II:** Comparison of biochemical parameters of groups by using Kruskal-Wallis Test.

Parameters	Group A* (n=52)	Group B** (n=69)	Group C*** (n=47)	p-value ^#^
Alkaline phosphatase (Units/L)	215.00 (179.00-251.00)	241.00 (201.50-280.50)	248.00 (176.00-283.00)	0.230
Calcium (mg/dl)	9.4 (8.90-10.00)	10.40 (9.55-11.45)	9.90 (9.40-10.40)	0.000^#^
Phosphate (mmol/L)	1.75 (1.27-2.20)	2.10 (1.25-2.60)	2.10 (1.60-2.32)	0.256

* Group A = Postmenopausal non-osteoporotic females

** Group B = Postmenopausal osteopenic females

*** Group C = Postmenopausal osteoporotic females Values are given as Median (IQR).

# p-value ≤ 0.05 is considered statistically significant. Alpha 0.05 with Confidence Interval 95%

Alkaline phosphatase, calcium and phosphate were used in a stepwise multiple regression analysis to predict T-score in normal, osteopenic and osteoporotic postmenopausal females. In normal postmenopausal females, the prediction model contained two of the three predictors and was reached in two steps with no variables removed. The model was statistically significant, F(2, 41) = 6.041, p < 0.05, and accounted for approximately 22% of the variance of T-score (R^2^ = 0.228, Adjusted R^2^ = 0.190). T-score was primarily predicted by higher levels of phosphate and calcium. The raw and standardized regression coefficients of predictors together with their correlations with T-score and their squared semipartial correlations are shown in Table-III. Phosphate received the strongest weight in the model followed by calcium. Phosphate and calcium uniquely accounted for approximately 19% and 10% of the variance of T-score.

**Table-III:** Stepwise regression results of postmenopausal normal and osteopenic females.

Coefficients		Model	Unstandardized Coefficients		Standardized Coefficients	t	Sig.	Correlations		
	
			B	Std. Error	Beta			Partial	Part	sr^2^
Group A	1	(Constant)	-.783	.169		-4.644	.000			
		Phosphate in mmol/l	.197	.080	.357	2.474	.017	.357	.357	
	2	(Constant)	-1.830	.481		-3.804	.000			
		Phosphate in mmol/l	.250	.079	.452	3.155	.003	.442	.433	.187
		Calcium in mg/dl	.100	.043	.331	2.308	.026	.339	.317	.100
Group B	1	(Constant)	-1.394	.275		-5.062	.000			
		Alkaline phosphatase in Units/liter	-.002	.001	-.279	-2.235	.029	-.279	-.279	.077

Dependent Variable: T-score

sr^2^ is the squared semi-partial correlation

Group A ◊ postmenopausal normal females,

Group B ◊ postmenopausal osteopenic females Read phonetically

In postmenopausal osteopenic females, the prediction model contained only one of the three predictors and was reached in one step with no variables removed. The model was statistically significant, F(1, 59) = 4.995, p < 0.05, and accounted for approximately 7% of the variance of T-score (R^2^ = 0.078, Adjusted R^2^ = 0.062). T-score was only predicted by lower levels of alkaline phosphatase. The raw and standardized regression coefficient of predictor together with its correlation with T-score and squared semipartial correlation is shown in Table-III. Alkaline phosphatase received the strongest weight in the model. In postmenopausal osteoporotic females, the prediction model contained no predictors.

## DISCUSSION

The loss of BMD in osteopenic group was supported by the increased activity of alkaline phosphatase and significantly raised serum calcium levels in osteopenic group. The normal range of calcium is 8.4-10.2 mg/dl. In our study serum calcium level was noted to be 10.40mg/dl in osteopenic group. That is slightly higher than the normal levels. which coincided with previous studies and suggested that towards menopause decalcification of bone starts due to which serum calcium level increases swiftly and in 2–5 years postmenopausally it crests, ebbing slightly afterwards as decalcification progresses.^15^ Stepwise multiple regression analysis for the prediction of T-score in all the three groups using alkaline phosphatase, calcium and phosphate has also showed that calcium and phosphate are the strongest predictors of T-score in postmenopausal normal females, while in postmenopausal osteopenic females alkaline phosphatase was the strongest predictor of T-score, that is higher levels of alkaline phosphatase were related with reduced T-scores which showed an imbalance between osteoblastic and osteoclastic activity shifting the equilibrium towards increase osteoclastic activity in osteopenic females that raises the levels of alkaline phosphatase in serum. Biver, et al. has also showed raised levels of bone alkaline phosphatase in osteoporotic patients compared to normal, and these high levels may be associated with prevalent vertebral fractures.^16^ Similarly, another study showed a strong negative relation between alkaline phosphatase and bone density in a stepwise regression analysis.^17^ In a recently conducted study ALP was found to be a predictor of BMD in end stage renal disease patients.^18^ So, evaluating alkaline phosphatase and other bone turn over markers may help in determining loss of bone mineral density in postmenopausal females.^19^-^21^ In another interesting study the researcher found that salivary calcium and alkaline phosphatase are also raised in osteopenic and osteoporotic patients and thus can be used for the diagnosis of underlying bone disorders^22^ It is important to note that multiple factors affects bone density.^23^,^24^ These effects influence bone through complex pathways. Serum levels of alkaline phosphatase could also be used as an index of decrease in bone mineral density. Further longitudinal studies are recommended to establish the role of these parameters in postmenopausal females as well as in males.

### Authors’ Contribution

***Sundus Tariq:*** Designed the study, data entry, manuscript writing.

***Saba Tariq:*** Data Collection and analysis, drafted the manuscript.

***Khalid Parvez Lone:*** Review and final approval of the manuscript.

***Saba Khaliq:*** Statistical analysis and edited the final manuscript.
